# Successful management of penetrating cardiac injury in a limited resource setting without cardiac surgical instruments and heart-lung machine

**DOI:** 10.1093/jscr/rjad473

**Published:** 2023-08-14

**Authors:** Ganesh Kumar K Ammannaya

**Affiliations:** Department of Cardiovascular & Thoracic Surgery, Accord Multispeciality Hospital, Bhuj, Gujarat 370001, India

## Abstract

Penetrating cardiac injuries (PCI) are often fatal and do not present enough time for effective referrals to higher centers. Most deaths occur in transit from a remote healthcare setting with limited resources. I present the first reported case of PCI in the medical literature to be managed successfully in the absence of heart-lung machine as well as dedicated cardiac surgical instruments and equipment, and which was further complicated by mediastinitis.

## INTRODUCTION

Penetrating cardiac injury (PCI) is one of the most lethal forms of chest trauma with mortality rate of around 16%–43% even if treated at level-1 quaternary centers [1, 2]. Hemorrhage, cardiac tamponade, and cardiac failure are the three common causes of fatality after PCI, cardiac tamponade being an early opportunity for survival, although contributing to rapid mortality. Emergent management is crucial for patients presenting with PCI, the definitive treatment being surgical drainage of pericardial blood and repair of underlying cardiac injury [2].

Mine is the first report in the medical literature till date of a PCI managed surgically without the use of any dedicated cardiac surgical instruments or equipment. Furthermore, the subsequent complications of sternal wound infection as well as mediastinitis were successfully managed with vacuum-assisted closure (VAC) dressings. Hence, this experience may serve as a guiding example for all resource-limited settings that may encounter PCIs but cannot risk referral of a collapsing patient to far flung centers.

## CASE REPORT

**Figure 1:**
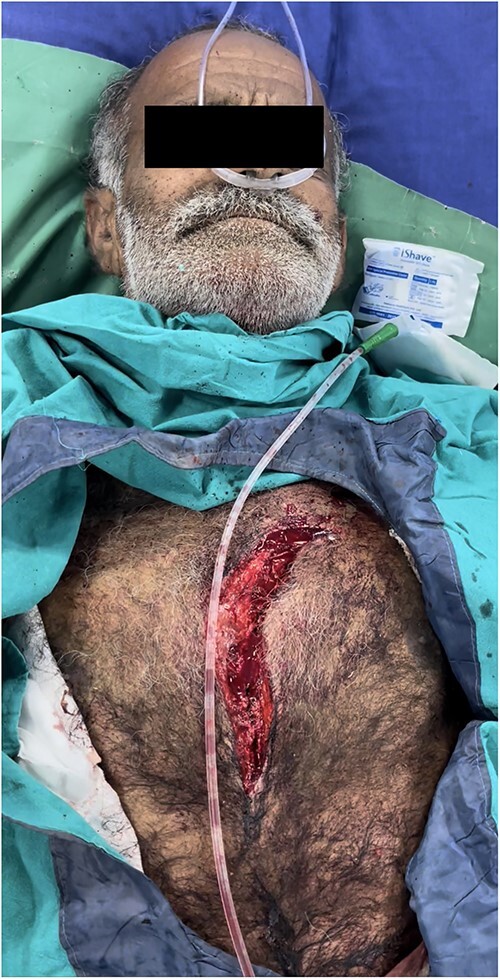
Penetrating chest injury at presentation.

A 63-year-old male presented to my new peripheral cardiac center, which was still being set up, with penetrating traumatic injury of heart and left lung resulting from a wood cutter accident (Fig. 1). He presented with severe shock with blood pressure of 70 mm Hg systolic in a semi-conscious state. Despite lack of equipment, dedicated cardiac surgical instruments, and heart-lung machine, decision to perform emergency exploration with cardiorrhaphy was taken as the nearest cardiac center was 5 h away.

**Figure 2:**
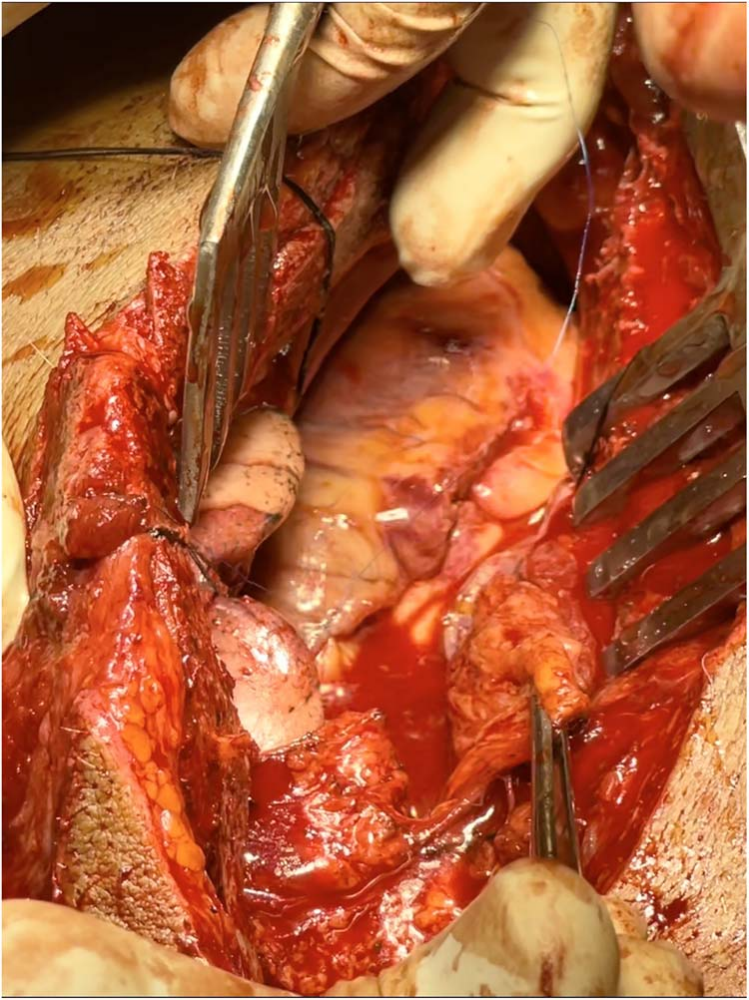
Intraoperative findings (sternal retraction with mastoid retractor)—hemopericardium with full thickness RVOT tear and partial thickness RV laceration.

**Figure 3:**
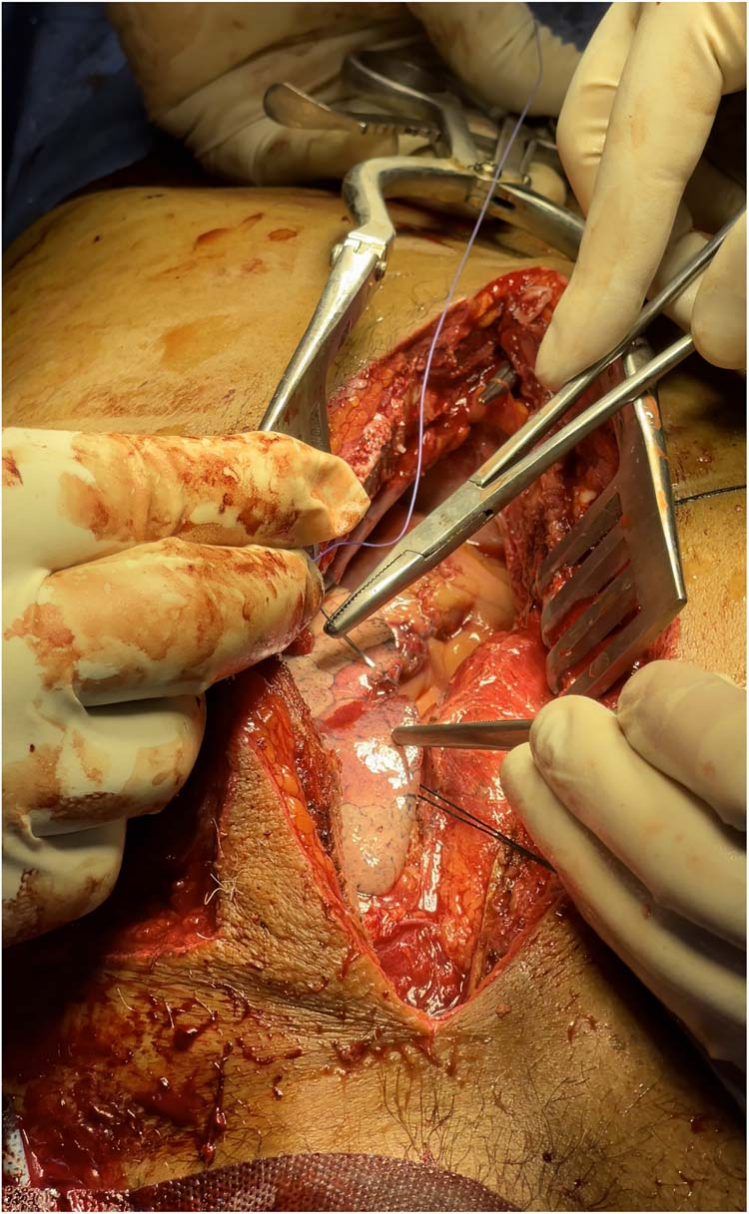
Repair of lung laceration.

**Figure 4:**
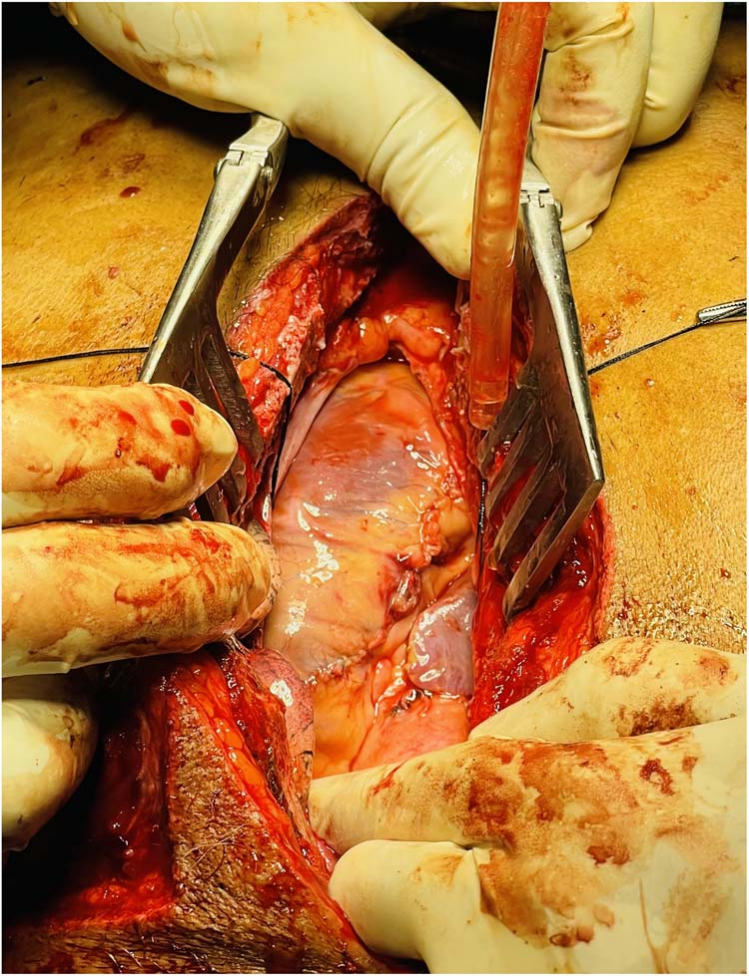
Repair of RVOT tear and partial thickness RV laceration.

A full median sternotomy approach was adopted, as a large sternal fracture was already present because of the accident. Sternotomy was performed with a neurosurgical craniotomy device, as a dedicated sternal saw was not available. Intraoperative findings included hemopericardium with full thickness right ventricular outflow tract (RVOT) tear of 2 cm length, a partial thickness right ventricular (RV) laceration of 5 cm length (Fig. 2), consistent with Grade IV cardiac injury (Organ Injury Scaling of the American Association for the Surgery of Trauma); and 3 × 2 cm lung laceration in the anterior margin of left upper lobe (Fig. 3). Both full thickness RVOT tear and partial thickness RV laceration were repaired meticulously with polypropylene 6–0 suture (Fig. 4) on the beating heart without any tissue stabilizer. Lung laceration was repaired in air tight manner with vicryl 3–0. Patient received a total of six units of packed red blood cells (PRBCs) intraoperatively. Chest closure was done with K-wire as standard steel wires were not available. Also, the left third and fourth rib fractures at the cartilaginous part were fixed with K-wires after placement of left pleural and mediastinal drains.

**Figure 5:**
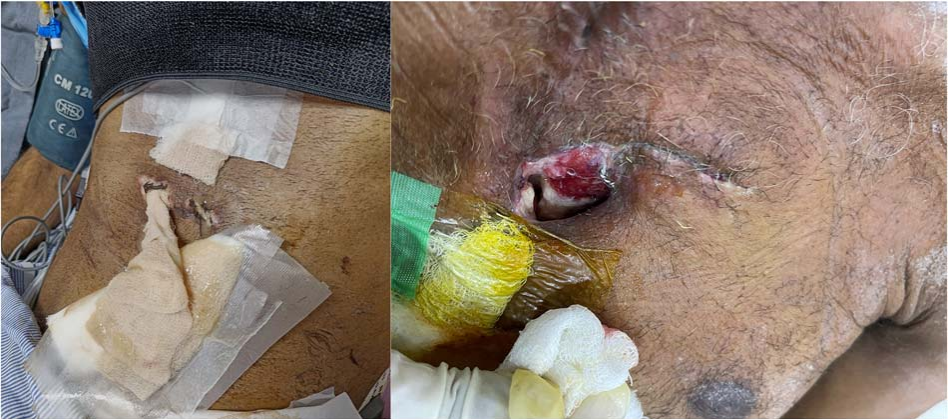
Wound infection with subsequent development of sternal instability and mediastinitis.

**Figure 6:**
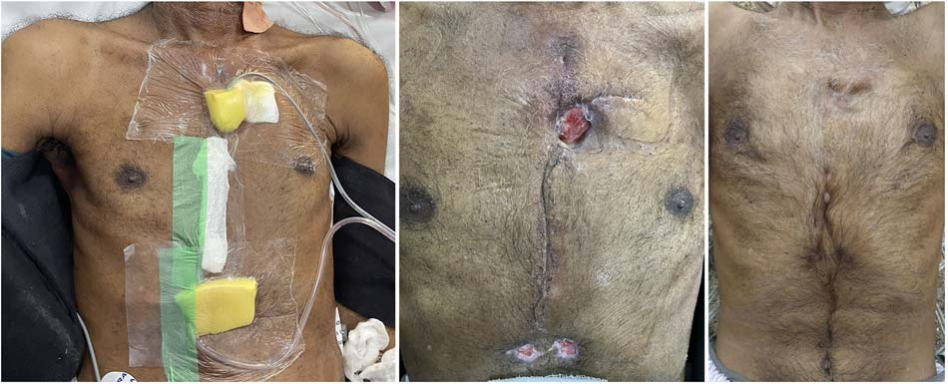
VAC dressing on Day 17; wound at discharge on Day 30; and at Day 60.

Patient was extubated the next day and had his chest drains removed by 3rd postoperative day. However, he developed post-cardiac injury syndrome (PCIS) on 5th postoperative day with bilateral pleural collection (right pleura was not opened during surgery), which prompted reinsertion of bilateral pleural drains. The course was further complicated by secondary infection of pleural effusion on 9th postoperative day needing continued drainage and iv antibiotics guided by sensitivity. He developed sternal wound infection on 11th postoperative day and sternal instability with mediastinitis on 17th postoperative day (Fig. 5). VAC dressing was commenced with the use of −150 mm Hg pressure and continued from 17th to 30th postoperative days, which enabled prompt resolution of mediastinitis enabling discharge of the patient on the 30th postoperative day by when sternal stability was achieved. VAC dressing was, however, continued till 60th postoperative day with home suction machine (alternate hourly suction applied), with weekly dressing changes. Compete wound healing was achieved by 60th postoperative day (Fig. 6).

## DISCUSSION

PCI is associated with an extremely high mortality rate and very few make it alive to the hospital. In a large series of 1198 patients with PCI in South Africa, merely 6% of patients arrived alive at a hospital [3]. While surgically managing such critical cases, it would always be highly recommended to have on-site facility of cardiopulmonary bypass (CPB), which may be lifesaving in case of any uncontrollable hemorrhage. Furthermore, mediastinitis and deep sternal wound infection (DSWI) are devastating and life-threatening complications of any cardiac surgery that is associated with a significantly high mortality rate of 10%–47% [4].

This is not only the first reported case in the medical literature of cardiac trauma that was operated without dedicated cardiac surgical instruments such as sternal spreader, tissue stabilizers, equipment such as sternal saw, standard sternal wires, and heart-lung machine, but also with the successful management of dreaded complications of DSWI and mediastinitis by VAC dressings after successful surgery.

## CONCLUSION

Successful management of PCI is achievable by the cardiothoracic surgeon in a limited resource setting despite a complicated postoperative course such as mediastinitis.
